# Independent trajectories of cognitive function and daily events across 9 years during midlife and old age

**DOI:** 10.1093/geronb/gbag122

**Published:** 2026-06-30

**Authors:** Patrick Klaiber, David M Almeida, Nicola Ballhausen

**Affiliations:** Department of Developmental Psychology, Tilburg University, Tilburg, The Netherlands; Department of Human Development and Family Studies, Pennsylvania State University, University Park, Pennsylvania, United States; Department of Developmental Psychology, Tilburg University, Tilburg, The Netherlands; (Psychological Sciences Section)

**Keywords:** Uplifts, Positive events, Hassles, Stressors, Stressful events

## Abstract

**Objectives:**

Daily positive and stressful events are determinants of everyday well-being, yet little is known about how they shift alongside normative cognitive aging. Losses in executive functioning and episodic memory may come along with a reduction in daily life engagement, such as the extent to which people experience positive events and stressors. Thus, we examined whether a 9-year change in cognitive functioning tracked with contemporaneous change in daily events.

**Methods:**

988 adults from the Midlife in the United States Study (baseline mean age 53.7 years) completed telephone assessments of executive functioning and episodic memory, and eight consecutive days of diaries assessing the occurrence of daily events at two waves about 9 years apart. Using structural equation models, we estimated mean change and co-change across cognition and daily positive events, and stressors.

**Results:**

On average, executive functioning showed declines in the overall sample, whereas episodic memory only showed declines among the older participants. Positive events increased while stressors slightly decreased. There was a positive association between changes in positive events and stressors: people who experienced an increase in positive events also experienced an increase in the number of stressors. Changes in cognition, however, were unrelated to changes in either event type.

**Discussion:**

In this population-based sample, normative cognitive aging appeared largely decoupled from concurrent shifts in daily affective events, which suggests that age-related changes in the frequency of daily events and cognition are driven by different underlying mechanisms.

## Graphical Abstract

**Graphical Abstract:**
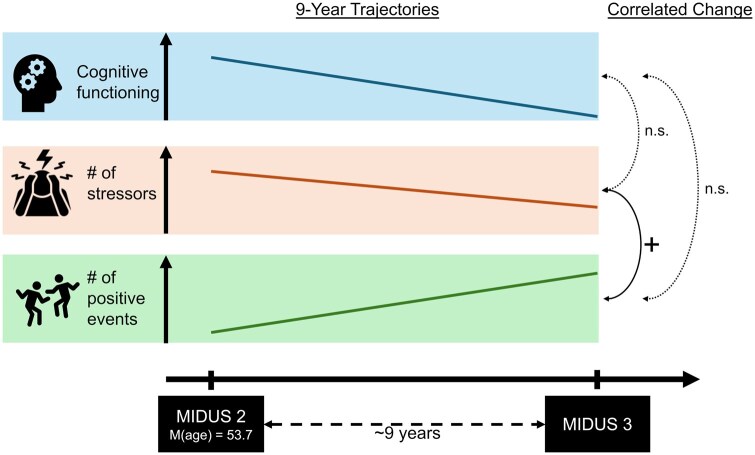

Executive functioning and episodic memory begin to display subtle declines in midlife (Karlamangla et al., 2017; Singh-Manoux et al., 2012). While initially modest, these changes tend to accelerate in older age (Rönnlund et al., 2005; Schaie & Willis, 2010). Importantly, there are large individual differences in these trajectories: some adults experience greater losses, whereas others maintain relatively stable levels well into old age (Nyberg et al., 2012; Stinchcombe et al., 2023). Overall, cognitive aging manifests as a gradual change, yet we still know little about whether these changes co-occur with changes in people’s everyday positive and negative experiences.

Daily events are important determinants of well-being. They reflect active engagement with the environment, capturing experiences that provide reinforcement, as well as daily demands that require adjustment (Klaiber et al., 2021). Positive events are minor, favorable transactions with the environment that usually result in upticks in positive emotions (Klaiber et al., 2022). Stressors, in contrast, encompass the routine challenges of everyday life, such as arguments, unexpected disruptions, and ongoing chronic strains (Almeida, 2005). Importantly, unlike perceived stress or feelings of stress, stressful events are external to a person and represent occurrences and conditions in the environment that place demands on the individual (Almeida, 2005). Although both positive events and stressors are thought to change in their frequency with age (Charles, 2010; Sin & Almeida, 2018), the evidence remains mixed, and little is known about how such shifts unfold in tandem with cognitive aging.

According to socioemotional selectivity theory, as people grow older, they increasingly prioritize emotionally meaningful relationships and activities while avoiding situations that might generate stress (Allen & Windsor, 2019; Carstensen et al., 1999; Sin & Almeida, 2018). By steering clear of potentially stressful contexts, however, older adults may also limit their opportunities for positive experiences. Indeed, longitudinal data from the VA Normative Aging Study have shown that exposure to both stressors and positive events decreased with advancing age (Aldwin et al., 2014). While this longitudinal pattern has been corroborated for stressors using the Midlife in the United States Study (MIDUS; Almeida et al., 2023), other longitudinal findings for positive events are missing. Cross-sectional studies are mixed, with studies focusing on older adulthood showing older age being related to fewer positive events (Charles et al., 2010; Klaiber & Pauly, 2025), while lifespan samples tend to show the opposite effect (Klaiber et al., 2021; Sin & Almeida, 2018). Importantly, these findings reflect average trends; there are marked individual differences in both the rate and direction of age-related change (Jeong et al., 2016).

Although both cognition and daily events change across midlife and older adulthood, it remains unclear whether these changes co-occur within individuals. Prior cross-sectional work has shown that individuals with better cognitive abilities report more frequent exposure to daily stressors (Stawski et al., 2013), possibly due to leading busier lives with more challenging careers. However, on the within-person level reflecting short-term fluctuations, stressors have been shown to hamper cognitive functioning, with people reporting more memory lapses and performing worse on cognitive tasks on days with a stressor, which has been explained by coping efforts depleting cognitive resources that are no longer available for other tasks (Saruhanlioglu et al., 2026; Sliwinski et al., 2006). Thus, over the long term, cumulative high exposure to stressful events may erode cognitive functioning and accelerate cognitive aging (Franks et al., 2023).

Evidence on links between daily positive events and cognition is more scarce, but better cognitive functioning was linked to more frequent positive events in both community and clinical samples cross-sectionally (Logsdon & Teri, 1997; Tse et al., 2024). While these findings highlight associations between daily experiences and cognition, most studies are cross-sectional, focus on pathological decline, or examine short-term covariation. Little is known about whether long-term changes in cognition and daily events are interrelated in community samples.

Theoretical perspectives suggest that long-term changes in cognition and daily experiences may be dynamically linked, rather than unfolding as independent processes. One source of this linkage is how individuals interpret their own cognitive aging: perceiving age-related decline can activate negative age stereotypes that dampen motivation to engage in everyday activities (Diehl & Wahl, 2024). The selection–optimization–compensation model (Baltes & Baltes, 1990) also proposes that lower cognitive resources increase selectivity, not only by discouraging cognitively demanding or stressful situations but by optimizing engagement in a smaller set of meaningful positive activities. Although adaptive, this narrowing may reduce both stressor exposure and engagement in positive events. Conversely, frequent participation in cognitively and socially stimulating activities may help maintain cognitive functioning (Fernández et al., 2023).

In this study, we leverage data from Waves 2 (M2) and 3 (M3) of the MIDUS Study, combining the cognitive and daily diary projects to examine 9-year changes in daily event exposure and cognitive functioning. Prior research in this sample has documented declines in cognitive abilities and stressor exposure (Almeida et al., 2023; Hughes et al., 2018). However, changes in positive events and, critically, whether changes in daily events and cognition co-occur, have not yet been investigated. Thus, we examine (a) whether changes in daily event exposure are associated with changes in executive functioning and episodic memory, and (b) whether baseline levels of daily events or cognition predict changes in either domain. This can clarify whether reductions in daily events are a side effect of normative cognitive aging, such as withdrawal from cognitively demanding situations, or whether changes in cognition and daily events reflect independent processes. In addition, we examine whether these associations vary by age. Aging-related changes often accelerate in older age (Rönnlund et al., 2005), and motivational theories of aging suggest that developmental processes are shaped by future time perspective (Carstensen et al., 1999), which becomes more limited with advanced age. Therefore, we expect older participants to show greater changes across the 9-year period than younger participants. We further expect that changes in daily events and cognition will be more strongly coupled among older participants.

## Method

### Procedure and sample

MIDUS is a longitudinal study of U.S. adults. The initial probability sample was recruited in 1995–1996. A subset completed the daily diary and cognitive projects. The cognitive project assessed executive functioning and episodic memory (M2: 2004–2006; M3: 2013–2017). The daily diary project consisted of up to eight telephone interviews on consecutive days regarding people’s same-day experiences (M2: 2004–2009, M3: 2017–2019; average completion rate = 7.5/8).

For demographic information and key study descriptives, see Table 1. Some selective dropout was evident, such that participants who completed the M3 cognitive assessment were younger, better educated, healthier, and performed better on all cognitive measures at M2 compared to drop-outs (Hughes et al., 2018).

**Table 1:** Descriptive statistics of all key study variables.

Variable	M2	M3
	*M* (*SD*) or %	*M* (*SD*)
**Demographics**		
Age in years (range: 34–81)	53.8 (10.15)	
Gender (% women)	57.3%	
Racial identity (% White)	86.8%	
Education (% with 4-year college degree)	44.2%	
**Episodic memory indicators^a^**		
Immediate word list recall	7.03 (2.16)	6.95 (2.31)
Delayed word list recall	4.81 (2.47)	4.60 (2.62)
**Executive functioning indicators^a^^,^^b^**		
Digits backward	5.01 (1.43)	4.99 (1.45)
Number series	2.48 (1.54)	2.35 (1.56)
Category fluency	20.03 (6.02)	19.48 (5.75)
Backward counting	39.30 (11.30)	37.00 (11.64)
Stop & Go	1.06 (0.21)	1.36 (0.31)
**Daily event frequency^c^**		
Number of stressors (*M* per day)	0.58 (0.47)	0.51 (0.47)
Number of positive events (*M* per day)	1.17 (0.69)	1.49 (0.75)

*Note.* *M* = mean; *SD* = standard deviation. M2 and M3 refer to waves 2 and 3 of the MIDUS study. Values shown are raw (unstandardized) scores. Higher values indicate better performance for all indicators except Stop & Go, where higher (slower) latencies indicate poorer performance (Stop & Go was reverse-scored prior to inclusion in the models). The cognitive indicators were rescaled prior to analyses by dividing the values by the baseline *SD* to foster model convergence.

a Possible ranges: immediate and delayed word-list recall, 0–15 words; backward digit span, 2–8 (longest span achieved); number series, 0–5 correct.

b Category fluency (unique animals named in 60s) and backward counting (100–lowest number reached–errors, over 30s) are open-ended counts with no fixed ceiling. Stop & Go is the mean response latency in seconds.

c Daily event frequency refers to the average daily number of event categories endorsed in the MIDUS daily diary project. Possible ranges: stressors, 0–7; positive events, 0–5.

### Measures

#### Brief test of adult cognition (BTACT)

Episodic memory and executive functioning were assessed with the BTACT, a validated instrument developed for large-scale surveys and longitudinal studies of midlife and aging (for a full description and psychometric properties, see Lachman et al., 2014). Episodic memory was measured with immediate and delayed free recall of a 15-word list (i.e., 0–15 for each trial). The number of correctly recalled words on the immediate and delayed trials served as two separate manifest indicators of episodic memory. Executive functioning was evaluated with five tasks, including one working memory task, which were shown to load on the same underlying factor (Lachman et al., 2014). (1) Category verbal fluency was assessed by counting the number of unique animal names generated in 60 seconds. (2) Inductive reasoning was assessed with a set of number series. The number of correctly completed numeric patterns out of 5 was used as an indicator. (3) Processing speed was assessed with a 30-second backward counting speed task, and the maximum number of correct items produced counting backward from 100, with errors subtracted, was used as an indicator. (4) A Stop & Go Switch Task was used to assess reaction time, attention, task switching, and inhibitory control. The mean response latency when switching between congruent and incongruent “stop” and “go” trials was used as an indicator. (5) Finally, a backward digit span task in which participants had to repeat a list of numbers in reverse order was used to assess working memory. The length of the highest span achieved (up to 8) was used as an indicator. For all cognitive indicators, higher values represent better performance with the exception of the stop-and-go task, which was reversed prior to the inclusion in the structural equation models. All five test scores were used as manifest indicators of executive functioning in the structural equation models after dividing each test score by its respective standard deviation at M2 to facilitate model convergence.

#### Daily positive events and stressors

Positive events and stressors were assessed with the Daily Inventory of Stressful Events (Almeida et al., 2002). Participants reported the occurrence of seven stressor types in the last 24 hours, which included (1) arguments or (2) avoided arguments, (3) work- or (4) home-related stressors, (5) experiences of discrimination, (6) negative events that happened to close others, and (7) other stressful events. Regarding positive events, participants reported whether they had experienced each of five types of positive events that day, including (1) positive social interactions, (2) events at work, school, or volunteering, (3) events at home, (4) positive events that happened to close others, or (5) other positive events. For the present study, we used the number of endorsed stressors and positive event categories each day, averaged across the diary period, as single manifest indicators in the structural equation models.

To see how representative the daily diary week was, participants were asked how stressful and positive this week was compared to their typical week. At M2, about two-thirds of participants said the diary week was about as stressful as a usual week, while 16% felt it was more stressful and 16% felt it was less stressful. For positive feelings, 88% of participants said they felt as positive as usual, while 7% said they felt positive more often, and 5% said they felt positive less often. Taken together, these results suggest that the 8-day assessment generally reflected a typical week in participants’ lives.

### Data analysis

**Figure 1:**
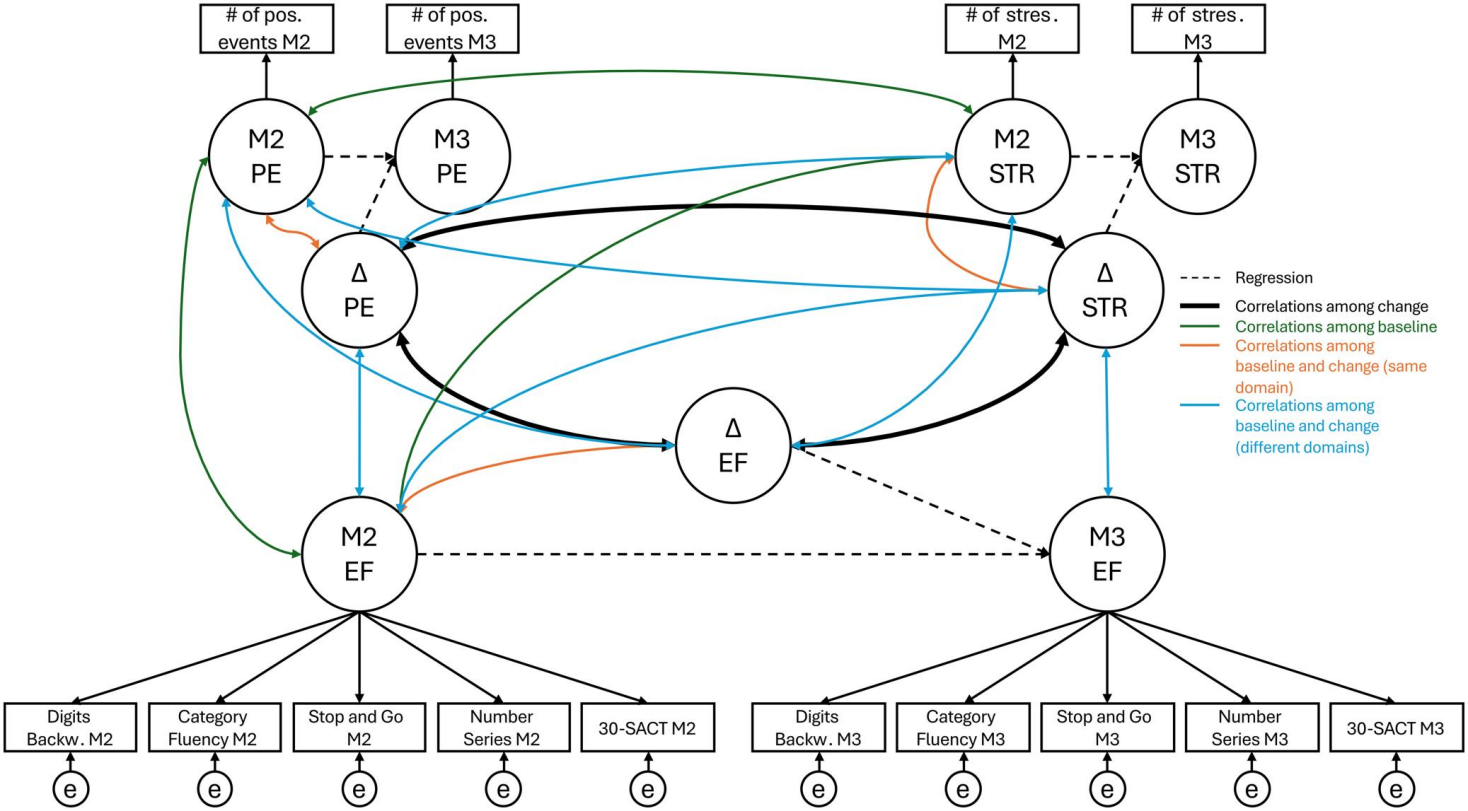
Correlated latent change model among executive functioning, positive events, and stressors. PE = positive events, STR = stressors, EF = executive functioning. Residuals of the same cognitive tasks were allowed to covary with themselves across time, intercepts, and loading were constrained to be equal across time; intercepts of the Stop & Go and 30-Seconds-And-Counting Task (30-SACT) were freed (partial scalar invariance). The model for episodic memory is included in Supplementary material.

We estimated two-wave latent change score models (LCSMs) in lavaan separately for executive functioning and episodic memory (Figure 1). Executive functioning was specified as a latent factor at each wave with equal factor loadings across waves and partial scalar invariance, whereas episodic memory was modeled using two indicators with factor loadings fixed to one and equal intercepts across waves; same-test residuals were correlated over time. Positive events and stressors were specified as single-indicator latent variables. For each domain, we applied an identity-form LCSM to estimate latent change and cross-domain covariation. We first fit unconditional models to examine raw level–change associations, followed by covariate-adjusted models including age at M2 (grand-mean-centered), number of chronic conditions at M2, race, education, and gender as predictors of both levels and changes. All models were estimated using robust maximum likelihood with full-information maximum likelihood to handle missing data. A detailed description of model specifications, estimation procedures, lavaan code, and full results is provided in Supplementary material and on the Open Science Framework (https://osf.io/dsrc5).

## Results

### Executive functioning

Table 2 displays the standardized model estimates. In the model without covariates, executive functioning declined by about half a standard deviation over the 9 years (dEF = –0.48). In contrast, positive events increased by about half a standard deviation (dPOS = 0.50), and stressors showed a slight decrease (dSTR = –0.15). In unstandardized terms, participants reported about one additional positive event every 3 days during M3 compared to M2 (dPOS = 0.32), whereas the decrease in stressors was relatively small (dSTR = –0.07), corresponding to about one fewer stressor per week. All latent change factors indicated substantial individual differences in trajectories.

**Table 2:** Standardized key parameters from latent change score models examining correlated change from MIDUS2 to MIDUS3.

Parameter	Executive functioning models		Episodic memory models	
	Without covariates	With covariates	Without covariates	With covariates
**Mean change**				
dEF/dEM	−0.479***	−0.626**	−0.060	−0.049
dPOS	0.496***	0.295**	0.496***	0.293**
dSTR	−0.150***	−0.033	−0.149***	−0.033
**Baseline correlations**				
EF/EM2∼∼POS2	0.145***	0.105*	0.125***	0.064
EF/EM2∼∼STR2	0.192***	0.113*	0.087**	−0.000
POS2∼∼STR2	0.444***	0.438***	0.444***	0.438***
**Correlated change**				
dEF/dEM∼∼dPOS	0.008	0.018	0.030	0.040
dEF/dEM∼∼dSTR	0.025	0.030	0.008	0.013
dPOS∼∼dSTR	0.136**	0.139**	0.136**	0.139**
**Correlations baseline with change**				
EF/EM2∼∼dEF/dEM	0.023	−0.114	−0.386***	−0.476***
EF/EM2∼∼dPOS	−0.010	−0.016	0.019	0.021
EF/EM2∼∼dSTR	−0.035	−0.044	−0.050	−0.033
POS2∼∼dPOS	−0.364***	−0.400***	−0.363***	−0.399***
POS2∼∼dEF/dEM	0.026	0.052	0.005	0.010
POS2∼∼dSTR	−0.165***	−0.158***	−0.164***	−0.157***
STR2∼∼dSTR	−0.481***	−0.504***	−0.482***	−0.504***
STR2∼∼dEF/dEM	−0.059	−0.076	0.072	0.065
STR2∼∼dPOS	−0.118**	−0.116**	−0.118**	−0.116**
**Effects of age on baseline and change parameters**				
Age → EF/EM2	—	−0.367***	—	−0.186***
Age → dEF/dEM	—	−0.220**	—	−0.116***
Age → POS2	—	0.072*	—	0.072*
Age → dPOS	—	0.084*	—	0.083*
Age → STR2	—	−0.185***	—	−0.185***
Age → dSTR	—	−0.009	—	−0.009

*Note.* EF = executive functioning, EM = episodic memory, POS = the number of positive events, STR = the number of stressors; d = change from M2 to M3; ∼∼ = correlations; → = regression paths. The full list of covariates includes age, number of chronic conditions, gender, education, and race. Full models with all parameter estimates can be found in Supplementary material.

* *p* < .05. **p < .01. ***p < .001.

Changes in executive functioning were unrelated to changes in positive events or stressors; correlations between change scores were close to zero. However, changes in positive events and stressors were positively related (correlation of change scores: *r* = 0.14), with individuals showing an increase in positive events also being more likely to show an increase in the number of stressors. Higher executive functioning at M2 was associated with reporting more positive events and stressors (baseline correlations). Similarly, people who reported more stressors at M2 also reported more positive events. Interestingly, M2 executive functioning was unrelated to changes in executive functioning, whereas higher M2 levels of positive events or stressors were negatively related to changes in both domains (baseline–change correlations).

Adding covariates (age, chronic conditions, gender, education, race) did not alter the pattern of correlations among the latent factors. The estimated means shifted slightly due to conditioning on covariates, but the substantive conclusions remained unchanged. Some additional effects of covariates emerged. Older participants had lower M2 executive functioning and greater decline but also reported more positive events at M2 and a greater increase in positive events. Older participants reported fewer stressors at M2, although age was unrelated to change in stressors. See Supplementary material for all covariate effects.

### Episodic memory

Results for episodic memory mirrored those for executive functioning, with several key differences. Episodic memory did not significantly change from M2 to M3 (dEM = –0.06, *p* = .09). M2 levels, however, were predictive: higher episodic memory at M2 was associated with greater subsequent decline (baseline change correlation: *r* = –0.39). Importantly, changes in episodic memory were unrelated to changes in either positive events or stressors (change-change correlations), as well as to their baseline levels (baseline-change correlation). Including covariates did not alter the main pattern of findings. Older participants performed worse on the memory task at M2 and showed a greater decline over time compared to younger participants.

## Discussion

This study found no evidence for correlated change between cognitive functioning and the frequency of daily events across midlife and old age. While individuals, on average, showed a decline in executive functioning, but not in episodic memory, they also reported an increase in the frequency of positive events and a decrease in stressors. Changes in cognition and events appeared to occur independently of one another. However, positive events and stressors were positively correlated both at baseline and in their longitudinal changes, indicating that individuals who experienced fewer stressors also tended to experience fewer positive events, and that reductions in stressors often coincided with reductions in positive events, perhaps reflecting shared changes in daily life engagement. Moreover, baseline levels of stressors and positive events were unrelated to subsequent cognitive changes, suggesting that positive experiences did not buffer, nor did stressors exacerbate, normative cognitive aging.

Overall, the results suggest that normative cognitive aging unfolds relatively independently of changes in daily life engagement, as indicated by the frequency of stressors and positive events. Whereas prior research has found that decreases in cognitive abilities are linked to reductions in positive affect (Castro-Schilo et al., 2019), the present findings indicate that this interdependence does not extend to the occurrence of positive events or stressors. Normative cognitive aging reflects gradual neurobiological changes, including cortical and white-matter decline, reduced dopaminergic function, and diminished neural efficiency (Raz & Rodrigue, 2006), that progress largely independently of day-to-day motivational regulation. In contrast, changes in daily events are likely shaped by adaptive motivational processes, including the avoidance of potentially stressful situations and the active pursuit of emotionally rewarding contexts (Carstensen et al., 1999; Charles, 2010). The absence of correlated change between cognitive and daily event trajectories is consistent with the notion that non-clinical biological and motivational aging processes operate in parallel yet largely distinct domains.

We also replicated findings that better cognitive functioning is associated with reporting more stressors and positive events in daily life (Stawski et al., 2013; Tse et al., 2024), potentially reflecting greater life engagement among cognitively higher-functioning individuals. A limitation of the present design is the mismatch in temporal resolution between assessments: whereas daily events were measured repeatedly over eight consecutive days, cognitive functioning was assessed only once per wave. This precludes conclusions about whether daily fluctuations in event exposure predict day-to-day variation in cognitive performance, or vice versa, and whether such dynamics differ across age groups. Intensive longitudinal designs combining daily diary methods with ambulatory cognitive assessment are needed to address these questions in future work.

In addition to event frequency, affective event reactivity may be more closely linked with changes in cognitive functioning. Individuals differ in their emotional reactions to stressors, which are meaningfully related to broader patterns of health and functioning. Stawski et al. (2010) found that people with higher fluid cognitive ability exhibited smaller increases in negative affect on stressor days, suggesting greater emotional regulation capacity in the face of everyday challenges. Thus, even if the *occurrence* of daily events is not correlated with cognitive trajectories, future research may examine whether individuals’ emotional responses to those events reflect cognitive aging processes.

There was also evidence for both age-related and cohort effects in the frequency of positive events. Over time, the number of positive events increased, and earlier-born cohorts not only reported more positive events overall but also showed greater increases. These findings align with socioemotional selectivity theory, which posits that with older age, individuals increasingly prioritize emotionally meaningful relationships and experiences (Carstensen et al., 1999). The cohort effects should be interpreted with caution, as they may partly reflect survivor bias, wherein less well-adjusted individuals from earlier-born cohorts are more likely to have dropped out of the study. Although some have argued that older adults’ avoidance of stressors might limit opportunities for positive experiences (Sin & Almeida, 2018), our findings suggest otherwise: older age was associated with more, not fewer, positive events. However, individuals who experienced reductions in stressors also tended to experience reductions in positive events, providing empirical support for the idea that lower engagement, manifesting as fewer stressors and positive events, may reflect a more reclusive lifestyle rather than an inevitable effect of aging.

Several limitations should be acknowledged. Participants who reported higher levels of positive events, stressors, or episodic memory at baseline showed greater subsequent declines, which may partly reflect regression to the mean. We were able to partially address this concern by using structural equation modeling, which accounts for measurement error in the BTACT through latent variable estimation, thereby reducing the degree to which unreliability at baseline could inflate apparent decline. Furthermore, daily event scores were based on multiple assessments at each wave on the specific day on which the events occurred, minimizing the risk of measurement error. In addition, the vast majority of participants indicated that their diary weeks were broadly typical of their daily lives, suggesting that baseline event counts were not driven by unusually eventful periods, which further reduces the likelihood that observed declines reflect a statistical artifact rather than a genuine change.

Moreover, the BTACT assesses normal cognitive aging and is not designed to detect cognitive pathology (Lachman et al., 2014). Accordingly, the present findings should be interpreted as reflecting normative aging, and results may differ in clinical populations. Selective attrition over the 9 years also likely excluded individuals who experienced more significant cognitive losses, among whom stronger associations between daily life and cognitive change might be expected.

In this national sample of U.S. adults, 9-year changes in executive functioning and episodic memory were unrelated to changes in daily positive events and stressors, suggesting that normative cognitive aging unfolds largely independently from everyday emotional engagement. Future studies should test whether stronger couplings emerge in at-risk or clinical populations and examine additional aspects of daily life, such as social interaction quality or affective reactivity, that may better capture how cognitive and motivational processes co-develop in adulthood.

## Supplementary material

Supplementary material is available at *The Journals of Gerontology, Series B: Psychological Sciences and Social Sciences* online.

## Supplementary Material

gbag122_Supplementary_Data

## Data Availability

Data and materials are accessible via ICPSR (https://www.icpsr.umich.edu/), and data analytical code is available on the Open Science Framework (https://osf.io/dsrc5/). The study was not preregistered.
