# Supplementary material for: Independent trajectories of cognitive function and daily events across 9 years during midlife and old age
Source: J Gerontol B Psychol Sci Soc Sci. 2026 Jun 30;81(9):gbag122. doi: 10.1093/geronb/gbag122 (PMC13439926; doi:10.1093/geronb/gbag122)
Supplement: Supplementary file 1 [file gbag122_supplementary_data.docx]

***The Journals of Gerontology, Series B: Psychological Sciences and Social Sciences* Supplementary Material: Klaiber, Almedia, & Ballhausen. Independent trajectories of cognitive function and daily events across nine years during midlife and old age.**

**Contents**

[1.	Description of data analytical approach	2](#_Toc233303911)

[2.	Depiction of correlated latent change score model for episodic memory	3](#_Toc233303912)

[3.	Assessment of chronic conditions	4](#_Toc233303913)

[4.	Full Model: Executive Functioning without covariates	5](#_Toc233303914)

[4.1.	Model specifications	5](#_Toc233303915)

[4.2.	Global fit indices	5](#_Toc233303916)

[4.3.	Factor loadings (std.all)	5](#_Toc233303917)

[4.4.	Regressions (std.all)	5](#_Toc233303918)

[4.5.	Variances & covariances (std.all)	6](#_Toc233303919)

[4.6.	Intercepts / means	7](#_Toc233303920)

[4.7.	R-squared for endogenous variables	7](#_Toc233303921)

[5.	Full Model: Executive Functioning with covariates	8](#_Toc233303922)

[5.1.	Model specifications	8](#_Toc233303923)

[5.2.	Global fit indices	8](#_Toc233303924)

[5.3.	Factor loadings (std.all)	8](#_Toc233303925)

[5.4.	Regressions (std.all)	8](#_Toc233303926)

[5.5.	Variances & covariances (std.all)	9](#_Toc233303927)

[5.6.	Intercepts / means	9](#_Toc233303928)

[5.7.	R-squared for endogenous variables	11](#_Toc233303929)

[6.	Full Model: Episodic Memory without covariates	12](#_Toc233303930)

[6.1.	Model specifications	12](#_Toc233303931)

[6.2.	Global fit indices	12](#_Toc233303932)

[6.3.	Factor loadings (std.all)	12](#_Toc233303933)

[6.4.	Regressions (std.all)	12](#_Toc233303934)

[6.5.	Variances & covariances (std.all)	13](#_Toc233303935)

[6.6.	Intercepts / means	14](#_Toc233303936)

[6.7.	R-squared for endogenous variables	14](#_Toc233303937)

[7.	Full Model: Episodic Memory with covariates	15](#_Toc233303938)

[7.1.	Model specifications	15](#_Toc233303939)

[7.2.	Global fit indices	15](#_Toc233303940)

[7.3.	Factor loadings (std.all)	15](#_Toc233303941)

[7.4.	Regressions (std.all)	16](#_Toc233303942)

[7.5.	Variances & covariances (std.all)	16](#_Toc233303943)

[7.6.	Intercepts / means	18](#_Toc233303944)

[7.7.	R-squared for endogenous variables	18](#_Toc233303945)

[8.	Discussion of covariate effects	19](#_Toc233303946)

[8.1.	Covariate Effects on Executive Functioning and Daily Events	19](#_Toc233303947)

[8.2.	Covariate Effects on Episodic Memory and Daily Events	19](#_Toc233303948)

# 1. Description of data analytical approach

We implemented two-wave latent change score models (LCSM) using lavaan. Separate models were run for executive functioning and episodic memory (see Figure 1 main text and section 2). Executive functioning was modeled as a latent factor at each wave with equal factor loadings across waves and partial scalar invariance. Episodic memory was based on two indicators with factor loadings fixed to one and intercepts to be equal across waves. Same-test residuals were correlated across waves. Positive events and stressors were specified as single-indicator latent variables with their factor loading fixed to one. For each domain (Cognition, Positive Events, Stressors) we used the identity-form LCSM: the M3 latent factor was regressed on the M2 latent factor with the regression coefficient fixed to one; a latent change factor was defined by fixing its loading to one on the M3 factor; the residual variance of the M3 factor was fixed to zero; the M2 latent mean was fixed to zero; and the mean of the change factor was freely estimated. We estimated covariances among all latent variables.

First, we fit unconditional models without covariates to quantify raw level–change and cross-domain coupling of changes. Second, as a sensitivity and adjustment step, we added covariates, grand-mean centered age at M2, grand-mean centered number of chronic conditions at M2, race (ref = non-white), education (ref = no college degree), and gender (ref = man), as predictors of both mean and change in all domains, yielding covariate-adjusted change factors and residual change variances. All models used robust maximum likelihood estimation with full-information maximum likelihood for missing data.

Lavaan code and full model results are accessible via the OSF: <https://osf.io/dsrc5/overview?view_only=33c130d03bfc43b0a6d3c429d5b5fedc>

# 2. Depiction of correlated latent change score model for episodic memory

*Note.* Residuals of same cognitive tasks were allowed to covary with themselves across time, loadings were fixed to 1, intercepts were constrained to be equal across time; PE = Positive Events, STR = Stressors, EM = Episodic Memory

# 3. Assessment of chronic conditions

Chronic conditions were assessed using a checklist of 30 health conditions from the Self-Administered Questionnaire (Section A, Question 11). Participants indicated which of the following conditions they had experienced or been treated for in the past 12 months: asthma, bronchitis, or emphysema; tuberculosis; other lung problems; arthritis, rheumatism, or other bone or joint diseases; sciatica, lumbago, or recurring backache; persistent skin trouble; thyroid disease; hay fever; recurring stomach trouble, indigestion, or diarrhea; urinary or bladder problems; being constipated all or most of the time; gall bladder trouble; persistent foot trouble; trouble with varicose veins requiring medical treatment; AIDS or HIV infection; lupus or other autoimmune disorders; persistent trouble with gums or mouth; persistent trouble with teeth; high blood pressure or hypertension; anxiety, depression, or some other emotional disorder; alcohol or drug problems; migraine headaches; chronic sleeping problems; diabetes or high blood sugar; multiple sclerosis, epilepsy, or other neurological disorders; stroke; ulcer; hernia or rupture; piles or hemorrhoids; and swallowing problems. The total number of chronic conditions was computed by summing all "Yes" responses, yielding a continuous score.

# 4. Full Model: Executive Functioning without covariates

## 4.1. Model specifications

| Specification | Value |
| --- | --- |
| Estimator |  |
| Missing handling | ml |
| # Groups | 1 |
| Group labels | (none) |
| N (total) | 988 |

## 4.2. Global fit indices

| Measure | Value |
| --- | --- |
| Chi-square | 165.892 |
| df | 67 |
| p-value | < .001 |
| CFI | 0.980 |
| TLI | 0.973 |
| RMSEA | 0.039 |
| RMSEA 90% CI lower | 0.031 |
| RMSEA 90% CI upper | 0.046 |
| RMSEA p-close |  |
| SRMR | 0.038 |
| AIC | 30581.080 |
| BIC | 30835.656 |

## 4.3. Factor loadings (std.all)

| Term | Est | SE | z-value | p | sig | CI | Std (all) |
| --- | --- | --- | --- | --- | --- | --- | --- |
| EF2 =~ M2 DIG BACKW | 1.000 | 0.000 |  |  |  | [1.000, 1.000] | 0.437 |
| EF2 =~ M2 CAT FLUEN | 1.102 | 0.101 | 10.966 | < .001 | *** | [0.905, 1.299] | 0.486 |
| EF2 =~ M2 STOP & GO | 1.285 | 0.138 | 9.294 | < .001 | *** | [1.014, 1.557] | 0.565 |
| EF2 =~ M2 NUM SERIES | 1.527 | 0.105 | 14.598 | < .001 | *** | [1.322, 1.732] | 0.669 |
| EF2 =~ M2 30S AND COUNT | 1.715 | 0.142 | 12.044 | < .001 | *** | [1.436, 1.994] | 0.753 |
| EF3 =~ M3 DIG BACKW | 1.000 | 0.000 |  |  |  | [1.000, 1.000] | 0.456 |
| EF3 =~ M3 CAT FLUEN | 1.102 | 0.101 | 10.966 | < .001 | *** | [0.905, 1.299] | 0.528 |
| EF3 =~ M3 STOP & GO | 1.285 | 0.138 | 9.294 | < .001 | *** | [1.014, 1.557] | 0.388 |
| EF3 =~ M3 NUM SERIES | 1.527 | 0.105 | 14.598 | < .001 | *** | [1.322, 1.732] | 0.695 |
| EF3 =~ M3 30S AND COUNT | 1.715 | 0.142 | 12.044 | < .001 | *** | [1.436, 1.994] | 0.764 |
| dEF =~ EF3 | 1.000 | 0.000 |  |  |  | [1.000, 1.000] | 0.273 |
| POS2 =~ pos_mean_dd2 | 1.000 | 0.000 |  |  |  | [1.000, 1.000] | 1.000 |
| POS3 =~ pos_mean_dd3 | 1.000 | 0.000 |  |  |  | [1.000, 1.000] | 1.000 |
| dPOS =~ POS3 | 1.000 | 0.000 |  |  |  | [1.000, 1.000] | 0.855 |
| STR2 =~ str_mean_dd2 | 1.000 | 0.000 |  |  |  | [1.000, 1.000] | 1.000 |
| STR3 =~ str_mean_dd3 | 1.000 | 0.000 |  |  |  | [1.000, 1.000] | 1.000 |
| dSTR =~ STR3 | 1.000 | 0.000 |  |  |  | [1.000, 1.000] | 0.962 |

## 4.4. Regressions (std.all)

| Term | Est | SE | z-value | p | sig | CI | Std (all) |
| --- | --- | --- | --- | --- | --- | --- | --- |
| EF3 ~ EF2 | 1.000 | 0.000 |  |  |  | [1.000, 1.000] | 0.956 |
| POS3 ~ POS2 | 1.000 | 0.000 |  |  |  | [1.000, 1.000] | 0.915 |
| STR3 ~ STR2 | 1.000 | 0.000 |  |  |  | [1.000, 1.000] | 1.000 |

## 4.5. Variances & covariances (std.all)

| Term | Est | SE | z-value | p | sig | CI | Std (all) |
| --- | --- | --- | --- | --- | --- | --- | --- |
| M2 DIG BACKW ~~ M3 DIG BACKW | 0.306 | 0.035 | 8.704 | < .001 | *** | [0.237, 0.375] | 0.378 |
| M2 CAT FLUEN ~~ M3 CAT FLUEN | 0.390 | 0.032 | 12.214 | < .001 | *** | [0.327, 0.452] | 0.548 |
| M2 STOP & GO ~~ M3 STOP & GO | 0.337 | 0.042 | 7.967 | < .001 | *** | [0.254, 0.419] | 0.290 |
| M2 NUM SERIES ~~ M3 NUM SERIES | 0.221 | 0.029 | 7.613 | < .001 | *** | [0.164, 0.278] | 0.407 |
| M2 30S AND COUNT ~~ M3 30S AND COUNT | 0.294 | 0.032 | 9.071 | < .001 | *** | [0.230, 0.357] | 0.669 |
| EF3 ~~ dEF | 0.000 | 0.000 |  |  |  | [0.000, 0.000] |  |
| Var(EF3) | 0.000 | 0.000 |  |  |  | [0.000, 0.000] | 0.000 |
| Var(EF2) | 0.193 | 0.029 | 6.732 | < .001 | *** | [0.137, 0.250] | 1.000 |
| Var(dEF) | 0.016 | 0.005 | 3.159 | .002 | ** | [0.006, 0.025] | 1.000 |
| EF2 ~~ dEF | 0.001 | 0.005 | 0.275 | .783 |  | [-0.008, 0.010] | 0.023 |
| POS3 ~~ dPOS | 0.000 | 0.000 |  |  |  | [0.000, 0.000] |  |
| Var(POS3) | 0.000 | 0.000 |  |  |  | [0.000, 0.000] | 0.000 |
| Var(POS2) | 0.470 | 0.024 | 19.410 | < .001 | *** | [0.422, 0.517] | 1.000 |
| Var(dPOS) | 0.410 | 0.023 | 18.050 | < .001 | *** | [0.365, 0.454] | 1.000 |
| POS2 ~~ dPOS | -0.159 | 0.017 | -9.214 | < .001 | *** | [-0.193, -0.126] | -0.364 |
| STR3 ~~ dSTR | 0.000 | 0.000 |  |  |  | [0.000, 0.000] |  |
| Var(STR3) | 0.000 | 0.000 |  |  |  | [0.000, 0.000] | 0.000 |
| Var(STR2) | 0.222 | 0.018 | 12.183 | < .001 | *** | [0.186, 0.258] | 1.000 |
| Var(dSTR) | 0.205 | 0.015 | 13.475 | < .001 | *** | [0.175, 0.235] | 1.000 |
| STR2 ~~ dSTR | -0.103 | 0.013 | -7.775 | < .001 | *** | [-0.129, -0.077] | -0.481 |
| EF2 ~~ POS2 | 0.044 | 0.012 | 3.755 | < .001 | *** | [0.021, 0.066] | 0.145 |
| EF2 ~~ STR2 | 0.040 | 0.008 | 4.826 | < .001 | *** | [0.024, 0.056] | 0.192 |
| POS2 ~~ STR2 | 0.143 | 0.014 | 10.030 | < .001 | *** | [0.115, 0.172] | 0.444 |
| dEF ~~ dPOS | 0.001 | 0.006 | 0.106 | .916 |  | [-0.012, 0.013] | 0.008 |
| dEF ~~ dSTR | 0.001 | 0.004 | 0.339 | .734 |  | [-0.007, 0.010] | 0.025 |
| dPOS ~~ dSTR | 0.040 | 0.012 | 3.178 | .001 | ** | [0.015, 0.064] | 0.136 |
| EF2 ~~ dPOS | -0.003 | 0.011 | -0.263 | .793 |  | [-0.024, 0.018] | -0.010 |
| EF2 ~~ dSTR | -0.007 | 0.008 | -0.875 | .381 |  | [-0.022, 0.009] | -0.035 |
| dEF ~~ POS2 | 0.002 | 0.006 | 0.366 | .714 |  | [-0.010, 0.014] | 0.026 |
| POS2 ~~ dSTR | -0.051 | 0.012 | -4.303 | < .001 | *** | [-0.074, -0.028] | -0.165 |
| dEF ~~ STR2 | -0.003 | 0.004 | -0.808 | .419 |  | [-0.012, 0.005] | -0.059 |
| dPOS ~~ STR2 | -0.036 | 0.013 | -2.818 | .005 | ** | [-0.060, -0.011] | -0.118 |
| Var(pos_mean_dd2) | 0.000 | 0.000 |  |  |  | [0.000, 0.000] | 0.000 |
| Var(pos_mean_dd3) | 0.000 | 0.000 |  |  |  | [0.000, 0.000] | 0.000 |
| Var(str_mean_dd2) | 0.000 | 0.000 |  |  |  | [0.000, 0.000] | 0.000 |
| Var(str_mean_dd3) | 0.000 | 0.000 |  |  |  | [0.000, 0.000] | 0.000 |
| Var(M2 DIG BACKW) | 0.818 | 0.045 | 17.973 | < .001 | *** | [0.728, 0.907] | 0.809 |
| Var(M2 CAT FLUEN) | 0.759 | 0.040 | 19.161 | < .001 | *** | [0.681, 0.837] | 0.764 |
| Var(M2 STOP & GO) | 0.681 | 0.048 | 14.304 | < .001 | *** | [0.588, 0.775] | 0.681 |
| Var(M2 NUM SERIES) | 0.558 | 0.033 | 16.852 | < .001 | *** | [0.493, 0.623] | 0.553 |
| Var(M2 30S AND COUNT) | 0.434 | 0.035 | 12.441 | < .001 | *** | [0.366, 0.502] | 0.433 |
| Var(M3 DIG BACKW) | 0.804 | 0.046 | 17.582 | < .001 | *** | [0.715, 0.894] | 0.792 |
| Var(M3 CAT FLUEN) | 0.666 | 0.036 | 18.308 | < .001 | *** | [0.595, 0.737] | 0.721 |
| Var(M3 STOP & GO) | 1.977 | 0.182 | 10.878 | < .001 | *** | [1.621, 2.333] | 0.850 |
| Var(M3 NUM SERIES) | 0.529 | 0.033 | 16.114 | < .001 | *** | [0.465, 0.594] | 0.517 |
| Var(M3 30S AND COUNT) | 0.444 | 0.040 | 11.142 | < .001 | *** | [0.366, 0.522] | 0.416 |

## 4.6. Intercepts / means

| Term | Est | SE | z-value | p | sig | CI | Std (all) |
| --- | --- | --- | --- | --- | --- | --- | --- |
| M2 DIG BACKW ~ 1 | 3.528 | 0.028 | 124.833 | < .001 | *** | [3.472, 3.583] | 3.509 |
| M3 DIG BACKW ~ 1 | 3.528 | 0.028 | 124.833 | < .001 | *** | [3.472, 3.583] | 3.500 |
| M2 CAT FLUEN ~ 1 | 3.314 | 0.029 | 113.569 | < .001 | *** | [3.256, 3.371] | 3.324 |
| M3 CAT FLUEN ~ 1 | 3.314 | 0.029 | 113.569 | < .001 | *** | [3.256, 3.371] | 3.449 |
| M2 NUM SERIES ~ 1 | 1.607 | 0.031 | 51.951 | < .001 | *** | [1.546, 1.668] | 1.600 |
| M3 NUM SERIES ~ 1 | 1.607 | 0.031 | 51.951 | < .001 | *** | [1.546, 1.668] | 1.589 |
| M2 30S AND COUNT ~ 1 | 3.475 | 0.032 | 108.956 | < .001 | *** | [3.412, 3.537] | 3.470 |
| M3 30S AND COUNT ~ 1 | 3.380 | 0.038 | 87.835 | < .001 | *** | [3.305, 3.455] | 3.273 |
| M2 STOP & GO ~ 1 | -5.104 | 0.032 | -158.478 | < .001 | *** | [-5.167, -5.041] | -5.102 |
| M3 STOP & GO ~ 1 | -6.500 | 0.051 | -128.505 | < .001 | *** | [-6.599, -6.401] | -4.262 |
| EF2 ~ 1 | 0.000 | 0.000 |  |  |  | [0.000, 0.000] | 0.000 |
| dEF ~ 1 | -0.060 | 0.014 | -4.412 | < .001 | *** | [-0.087, -0.033] | -0.479 |
| EF3 ~ 1 | 0.000 | 0.000 |  |  |  | [0.000, 0.000] | 0.000 |
| pos_mean_dd2 ~ 1 | 1.173 | 0.022 | 53.784 | < .001 | *** | [1.130, 1.215] | 1.711 |
| pos_mean_dd3 ~ 1 | 1.173 | 0.022 | 53.784 | < .001 | *** | [1.130, 1.215] | 1.566 |
| POS2 ~ 1 | 0.000 | 0.000 |  |  |  | [0.000, 0.000] | 0.000 |
| dPOS ~ 1 | 0.318 | 0.021 | 15.342 | < .001 | *** | [0.277, 0.358] | 0.496 |
| POS3 ~ 1 | 0.000 | 0.000 |  |  |  | [0.000, 0.000] | 0.000 |
| str_mean_dd2 ~ 1 | 0.579 | 0.015 | 38.605 | < .001 | *** | [0.549, 0.608] | 1.228 |
| str_mean_dd3 ~ 1 | 0.579 | 0.015 | 38.605 | < .001 | *** | [0.549, 0.608] | 1.229 |
| STR2 ~ 1 | 0.000 | 0.000 |  |  |  | [0.000, 0.000] | 0.000 |
| dSTR ~ 1 | -0.068 | 0.015 | -4.670 | < .001 | *** | [-0.096, -0.039] | -0.150 |
| STR3 ~ 1 | 0.000 | 0.000 |  |  |  | [0.000, 0.000] | 0.000 |

## 4.7. R-squared for endogenous variables

| Variable | R2 |
| --- | --- |
| EF3 | 1.000 |
| POS3 | 1.000 |
| STR3 | 1.000 |
| pos_mean_dd2 | 1.000 |
| pos_mean_dd3 | 1.000 |
| str_mean_dd2 | 1.000 |
| str_mean_dd3 | 1.000 |
| M2 DIG BACKW | 0.191 |
| M2 CAT FLUEN | 0.236 |
| M2 STOP & GO | 0.319 |
| M2 NUM SERIES | 0.447 |
| M2 30S AND COUNT | 0.567 |
| M3 DIG BACKW | 0.208 |
| M3 CAT FLUEN | 0.279 |
| M3 STOP & GO | 0.150 |
| M3 NUM SERIES | 0.483 |
| M3 30S AND COUNT | 0.584 |

# 5. Full Model: Executive Functioning with covariates

## 5.1. Model specifications

| Specification | Value |
| --- | --- |
| Estimator |  |
| Missing handling | ml |
| # Groups | 1 |
| Group labels | (none) |
| N (total) | 988 |

## 5.2. Global fit indices

| Measure | Value |
| --- | --- |
| Chi-square | 296.141 |
| df | 107 |
| p-value | < .001 |
| CFI | 0.968 |
| TLI | 0.949 |
| RMSEA | 0.042 |
| RMSEA 90% CI lower | 0.037 |
| RMSEA 90% CI upper | 0.048 |
| RMSEA p-close |  |
| SRMR | 0.036 |
| AIC | 45103.350 |
| BIC | 45602.709 |

## 5.3. Factor loadings (std.all)

| Term | Est | SE | z-value | p | sig | CI | Std (all) |
| --- | --- | --- | --- | --- | --- | --- | --- |
| EF2 =~ M2 DIG BACKW | 1.000 | 0.000 |  |  |  | [1.000, 1.000] | 0.424 |
| EF2 =~ M2 CAT FLUEN | 1.175 | 0.104 | 11.304 | < .001 | *** | [0.971, 1.379] | 0.503 |
| EF2 =~ M2 STOP & GO | 1.301 | 0.132 | 9.882 | < .001 | *** | [1.043, 1.559] | 0.556 |
| EF2 =~ B3TNSTOT | 1.612 | 0.111 | 14.478 | < .001 | *** | [1.394, 1.831] | 0.684 |
| EF2 =~ M2 30S AND COUNT | 1.739 | 0.131 | 13.267 | < .001 | *** | [1.482, 1.996] | 0.738 |
| EF3 =~ M3 DIG BACKW | 1.000 | 0.000 |  |  |  | [1.000, 1.000] | 0.442 |
| EF3 =~ M3 CAT FLUEN | 1.175 | 0.104 | 11.304 | < .001 | *** | [0.971, 1.379] | 0.544 |
| EF3 =~ M3 STOP & GO | 1.301 | 0.132 | 9.882 | < .001 | *** | [1.043, 1.559] | 0.379 |
| EF3 =~ C3TNSTOT | 1.612 | 0.111 | 14.478 | < .001 | *** | [1.394, 1.831] | 0.710 |
| EF3 =~ M3 30S AND COUNT | 1.739 | 0.131 | 13.267 | < .001 | *** | [1.482, 1.996] | 0.751 |
| dEF =~ EF3 | 1.000 | 0.000 |  |  |  | [1.000, 1.000] | 0.276 |
| POS2 =~ pos_mean_dd2 | 1.000 | 0.000 |  |  |  | [1.000, 1.000] | 1.000 |
| POS3 =~ pos_mean_dd3 | 1.000 | 0.000 |  |  |  | [1.000, 1.000] | 1.000 |
| dPOS =~ POS3 | 1.000 | 0.000 |  |  |  | [1.000, 1.000] | 0.858 |
| STR2 =~ str_mean_dd2 | 1.000 | 0.000 |  |  |  | [1.000, 1.000] | 1.000 |
| STR3 =~ str_mean_dd3 | 1.000 | 0.000 |  |  |  | [1.000, 1.000] | 1.000 |
| dSTR =~ STR3 | 1.000 | 0.000 |  |  |  | [1.000, 1.000] | 0.962 |

## 5.4. Regressions (std.all)

| Term | Est | SE | z-value | p | sig | CI | Std (all) |
| --- | --- | --- | --- | --- | --- | --- | --- |
| EF3 ~ EF2 | 1.000 | 0.000 |  |  |  | [1.000, 1.000] | 0.956 |
| POS3 ~ POS2 | 1.000 | 0.000 |  |  |  | [1.000, 1.000] | 0.917 |
| STR3 ~ STR2 | 1.000 | 0.000 |  |  |  | [1.000, 1.000] | 1.000 |
| EF2 ~ age_w2_c | -0.015 | 0.002 | -10.000 | < .001 | *** | [-0.018, -0.012] | -0.367 |
| EF2 ~ white | 0.340 | 0.047 | 7.277 | < .001 | *** | [0.248, 0.431] | 0.270 |
| EF2 ~ edu_college | 0.335 | 0.036 | 9.302 | < .001 | *** | [0.264, 0.405] | 0.390 |
| EF2 ~ sex_clean | -0.113 | 0.027 | -4.250 | < .001 | *** | [-0.166, -0.061] | -0.132 |
| EF2 ~ chronic_c | -0.023 | 0.006 | -3.754 | < .001 | *** | [-0.035, -0.011] | -0.123 |
| dEF ~ age_w2_c | -0.003 | 0.001 | -3.190 | .001 | ** | [-0.004, -0.001] | -0.220 |
| dEF ~ white | 0.028 | 0.026 | 1.094 | .274 |  | [-0.022, 0.079] | 0.078 |
| dEF ~ edu_college | -0.021 | 0.017 | -1.224 | .221 |  | [-0.054, 0.012] | -0.084 |
| dEF ~ sex_clean | 0.006 | 0.017 | 0.375 | .708 |  | [-0.027, 0.039] | 0.025 |
| dEF ~ chronic_c | -0.006 | 0.004 | -1.572 | .116 |  | [-0.014, 0.002] | -0.118 |
| POS2 ~ age_w2_c | 0.005 | 0.002 | 2.246 | .025 | * | [0.001, 0.009] | 0.072 |
| POS2 ~ white | 0.120 | 0.065 | 1.845 | .065 |  | [-0.007, 0.247] | 0.059 |
| POS2 ~ edu_college | 0.330 | 0.044 | 7.576 | < .001 | *** | [0.245, 0.416] | 0.239 |
| POS2 ~ sex_clean | 0.181 | 0.043 | 4.177 | < .001 | *** | [0.096, 0.266] | 0.131 |
| POS2 ~ chronic_c | -0.000 | 0.010 | -0.010 | .992 |  | [-0.019, 0.019] | -0.000 |
| dPOS ~ age_w2_c | 0.005 | 0.002 | 2.447 | .014 | * | [0.001, 0.009] | 0.084 |
| dPOS ~ white | 0.130 | 0.060 | 2.170 | .030 | * | [0.013, 0.248] | 0.069 |
| dPOS ~ edu_college | 0.018 | 0.045 | 0.409 | .683 |  | [-0.070, 0.106] | 0.014 |
| dPOS ~ sex_clean | 0.014 | 0.043 | 0.322 | .747 |  | [-0.070, 0.098] | 0.011 |
| dPOS ~ chronic_c | -0.008 | 0.010 | -0.777 | .437 |  | [-0.028, 0.012] | -0.028 |
| STR2 ~ age_w2_c | -0.008 | 0.001 | -6.176 | < .001 | *** | [-0.011, -0.006] | -0.185 |
| STR2 ~ white | 0.087 | 0.044 | 1.964 | .050 | * | [0.000, 0.173] | 0.062 |
| STR2 ~ edu_college | 0.187 | 0.032 | 5.861 | < .001 | *** | [0.125, 0.250] | 0.197 |
| STR2 ~ sex_clean | 0.080 | 0.029 | 2.738 | .006 | ** | [0.023, 0.138] | 0.084 |
| STR2 ~ chronic_c | 0.040 | 0.010 | 3.965 | < .001 | *** | [0.020, 0.059] | 0.192 |
| dSTR ~ age_w2_c | -0.000 | 0.002 | -0.250 | .803 |  | [-0.003, 0.003] | -0.009 |
| dSTR ~ white | -0.013 | 0.043 | -0.302 | .763 |  | [-0.098, 0.072] | -0.010 |
| dSTR ~ edu_college | -0.019 | 0.031 | -0.603 | .546 |  | [-0.079, 0.042] | -0.020 |
| dSTR ~ sex_clean | -0.059 | 0.030 | -1.926 | .054 |  | [-0.118, 0.001] | -0.064 |
| dSTR ~ chronic_c | 0.004 | 0.007 | 0.541 | .589 |  | [-0.011, 0.019] | 0.020 |

## 5.5. Variances & covariances (std.all)

| Term | Est | SE | z-value | p | sig | CI | Std (all) |
| --- | --- | --- | --- | --- | --- | --- | --- |
| M2 DIG BACKW ~~ M3 DIG BACKW | 0.319 | 0.034 | 9.383 | < .001 | *** | [0.253, 0.386] | 0.388 |
| M2 CAT FLUEN ~~ M3 CAT FLUEN | 0.373 | 0.030 | 12.299 | < .001 | *** | [0.314, 0.432] | 0.538 |
| M2 STOP & GO ~~ M3 STOP & GO | 0.345 | 0.042 | 8.201 | < .001 | *** | [0.263, 0.428] | 0.294 |
| B3TNSTOT ~~ C3TNSTOT | 0.201 | 0.026 | 7.779 | < .001 | *** | [0.150, 0.252] | 0.385 |
| M2 30S AND COUNT ~~ M3 30S AND COUNT | 0.319 | 0.029 | 10.878 | < .001 | *** | [0.261, 0.376] | 0.689 |
| EF3 ~~ dEF | 0.000 | 0.000 |  |  |  | [0.000, 0.000] |  |
| Var(EF3) | 0.000 | 0.000 |  |  |  | [0.000, 0.000] | 0.000 |
| Var(EF2) | 0.095 | 0.015 | 6.423 | < .001 | *** | [0.066, 0.124] | 0.524 |
| Var(dEF) | 0.014 | 0.005 | 3.031 | .002 | ** | [0.005, 0.023] | 0.922 |
| EF2 ~~ dEF | -0.004 | 0.003 | -1.192 | .233 |  | [-0.011, 0.003] | -0.114 |
| POS3 ~~ dPOS | 0.000 | 0.000 |  |  |  | [0.000, 0.000] |  |
| Var(POS3) | 0.000 | 0.000 |  |  |  | [0.000, 0.000] | 0.000 |
| Var(POS2) | 0.434 | 0.023 | 18.459 | < .001 | *** | [0.388, 0.480] | 0.923 |
| Var(dPOS) | 0.406 | 0.023 | 17.851 | < .001 | *** | [0.361, 0.450] | 0.987 |
| POS2 ~~ dPOS | -0.168 | 0.017 | -10.058 | < .001 | *** | [-0.201, -0.135] | -0.400 |
| STR3 ~~ dSTR | 0.000 | 0.000 |  |  |  | [0.000, 0.000] |  |
| Var(STR3) | 0.000 | 0.000 |  |  |  | [0.000, 0.000] | 0.000 |
| Var(STR2) | 0.199 | 0.016 | 12.381 | < .001 | *** | [0.167, 0.230] | 0.896 |
| Var(dSTR) | 0.204 | 0.015 | 13.610 | < .001 | *** | [0.175, 0.234] | 0.996 |
| STR2 ~~ dSTR | -0.102 | 0.012 | -8.127 | < .001 | *** | [-0.126, -0.077] | -0.504 |
| EF2 ~~ POS2 | 0.021 | 0.008 | 2.605 | .009 | ** | [0.005, 0.037] | 0.105 |
| EF2 ~~ STR2 | 0.016 | 0.006 | 2.546 | .011 | * | [0.004, 0.028] | 0.113 |
| POS2 ~~ STR2 | 0.129 | 0.013 | 10.144 | < .001 | *** | [0.104, 0.153] | 0.438 |
| dEF ~~ dPOS | 0.001 | 0.006 | 0.225 | .822 |  | [-0.011, 0.013] | 0.018 |
| dEF ~~ dSTR | 0.002 | 0.004 | 0.396 | .692 |  | [-0.006, 0.009] | 0.030 |
| dPOS ~~ dSTR | 0.040 | 0.012 | 3.245 | .001 | ** | [0.016, 0.064] | 0.139 |
| EF2 ~~ dPOS | -0.003 | 0.008 | -0.392 | .695 |  | [-0.019, 0.013] | -0.016 |
| EF2 ~~ dSTR | -0.006 | 0.006 | -1.065 | .287 |  | [-0.018, 0.005] | -0.044 |
| dEF ~~ POS2 | 0.004 | 0.006 | 0.706 | .480 |  | [-0.007, 0.015] | 0.052 |
| POS2 ~~ dSTR | -0.047 | 0.011 | -4.270 | < .001 | *** | [-0.068, -0.025] | -0.158 |
| dEF ~~ STR2 | -0.004 | 0.004 | -0.996 | .319 |  | [-0.012, 0.004] | -0.076 |
| dPOS ~~ STR2 | -0.033 | 0.012 | -2.691 | .007 | ** | [-0.057, -0.009] | -0.116 |
| Var(pos_mean_dd2) | 0.000 | 0.000 |  |  |  | [0.000, 0.000] | 0.000 |
| Var(pos_mean_dd3) | 0.000 | 0.000 |  |  |  | [0.000, 0.000] | 0.000 |
| Var(str_mean_dd2) | 0.000 | 0.000 |  |  |  | [0.000, 0.000] | 0.000 |
| Var(str_mean_dd3) | 0.000 | 0.000 |  |  |  | [0.000, 0.000] | 0.000 |
| age_w2_c ~~ white | 0.158 | 0.115 | 1.373 | .170 |  | [-0.067, 0.383] | 0.045 |
| age_w2_c ~~ edu_college | -0.216 | 0.163 | -1.322 | .186 |  | [-0.536, 0.104] | -0.042 |
| age_w2_c ~~ sex_clean | -0.017 | 0.162 | -0.102 | .919 |  | [-0.334, 0.301] | -0.003 |
| age_w2_c ~~ chronic_c | 3.801 | 0.738 | 5.150 | < .001 | *** | [2.354, 5.247] | 0.161 |
| white ~~ edu_college | 0.023 | 0.005 | 4.501 | < .001 | *** | [0.013, 0.033] | 0.137 |
| white ~~ sex_clean | -0.006 | 0.005 | -1.112 | .266 |  | [-0.016, 0.004] | -0.035 |
| white ~~ chronic_c | -0.044 | 0.028 | -1.603 | .109 |  | [-0.098, 0.010] | -0.057 |
| edu_college ~~ sex_clean | -0.033 | 0.008 | -4.298 | < .001 | *** | [-0.049, -0.018] | -0.136 |
| edu_college ~~ chronic_c | -0.145 | 0.035 | -4.118 | < .001 | *** | [-0.213, -0.076] | -0.128 |
| sex_clean ~~ chronic_c | 0.181 | 0.035 | 5.232 | < .001 | *** | [0.113, 0.249] | 0.161 |
| Var(M2 DIG BACKW) | 0.830 | 0.044 | 18.665 | < .001 | *** | [0.743, 0.917] | 0.821 |
| Var(M2 CAT FLUEN) | 0.738 | 0.037 | 19.700 | < .001 | *** | [0.665, 0.812] | 0.747 |
| Var(M2 STOP & GO) | 0.687 | 0.047 | 14.680 | < .001 | *** | [0.596, 0.779] | 0.691 |
| Var(B3TNSTOT) | 0.538 | 0.031 | 17.386 | < .001 | *** | [0.477, 0.599] | 0.533 |
| Var(M2 30S AND COUNT) | 0.460 | 0.032 | 14.393 | < .001 | *** | [0.398, 0.523] | 0.456 |
| Var(M3 DIG BACKW) | 0.817 | 0.045 | 18.117 | < .001 | *** | [0.729, 0.906] | 0.805 |
| Var(M3 CAT FLUEN) | 0.652 | 0.035 | 18.668 | < .001 | *** | [0.583, 0.720] | 0.704 |
| Var(M3 STOP & GO) | 2.003 | 0.183 | 10.927 | < .001 | *** | [1.644, 2.362] | 0.856 |
| Var(C3TNSTOT) | 0.507 | 0.029 | 17.361 | < .001 | *** | [0.450, 0.565] | 0.496 |
| Var(M3 30S AND COUNT) | 0.465 | 0.036 | 12.928 | < .001 | *** | [0.395, 0.536] | 0.436 |
| Var(age_w2_c) | 108.112 | 3.986 | 27.122 | < .001 | *** | [100.299, 115.925] | 1.000 |
| Var(white) | 0.115 | 0.008 | 14.421 | < .001 | *** | [0.099, 0.130] | 1.000 |
| Var(edu_college) | 0.247 | 0.002 | 134.669 | < .001 | *** | [0.243, 0.250] | 1.000 |
| Var(sex_clean) | 0.245 | 0.002 | 108.214 | < .001 | *** | [0.240, 0.249] | 1.000 |
| Var(chronic_c) | 5.159 | 0.448 | 11.506 | < .001 | *** | [4.280, 6.038] | 1.000 |

## 5.6. Intercepts / means

| Term | Est | SE | z-value | p | sig | CI | Std (all) |
| --- | --- | --- | --- | --- | --- | --- | --- |
| M2 DIG BACKW ~ 1 | 3.149 | 0.058 | 54.651 | < .001 | *** | [3.036, 3.262] | 3.130 |
| M3 DIG BACKW ~ 1 | 3.149 | 0.058 | 54.651 | < .001 | *** | [3.036, 3.262] | 3.124 |
| M2 CAT FLUEN ~ 1 | 2.871 | 0.061 | 46.776 | < .001 | *** | [2.750, 2.991] | 2.886 |
| M3 CAT FLUEN ~ 1 | 2.871 | 0.061 | 46.776 | < .001 | *** | [2.750, 2.991] | 2.984 |
| B3TNSTOT ~ 1 | 0.998 | 0.077 | 13.036 | < .001 | *** | [0.848, 1.148] | 0.993 |
| C3TNSTOT ~ 1 | 0.998 | 0.077 | 13.036 | < .001 | *** | [0.848, 1.148] | 0.986 |
| M2 30S AND COUNT ~ 1 | 2.818 | 0.082 | 34.543 | < .001 | *** | [2.658, 2.978] | 2.804 |
| M3 30S AND COUNT ~ 1 | 2.721 | 0.084 | 32.440 | < .001 | *** | [2.556, 2.885] | 2.635 |
| M2 STOP & GO ~ 1 | -5.596 | 0.078 | -72.002 | < .001 | *** | [-5.748, -5.444] | -5.611 |
| M3 STOP & GO ~ 1 | -6.994 | 0.086 | -81.741 | < .001 | *** | [-7.161, -6.826] | -4.573 |
| EF2 ~ 1 | 0.000 | 0.000 |  |  |  | [0.000, 0.000] | 0.000 |
| dEF ~ 1 | -0.077 | 0.029 | -2.682 | .007 | ** | [-0.133, -0.021] | -0.626 |
| EF3 ~ 1 | 0.000 | 0.000 |  |  |  | [0.000, 0.000] | 0.000 |
| pos_mean_dd2 ~ 1 | 0.819 | 0.067 | 12.239 | < .001 | *** | [0.688, 0.950] | 1.195 |
| pos_mean_dd3 ~ 1 | 0.819 | 0.067 | 12.239 | < .001 | *** | [0.688, 0.950] | 1.095 |
| POS2 ~ 1 | 0.000 | 0.000 |  |  |  | [0.000, 0.000] | 0.000 |
| dPOS ~ 1 | 0.189 | 0.062 | 3.036 | .002 | ** | [0.067, 0.311] | 0.295 |
| POS3 ~ 1 | 0.000 | 0.000 |  |  |  | [0.000, 0.000] | 0.000 |
| str_mean_dd2 ~ 1 | 0.375 | 0.046 | 8.106 | < .001 | *** | [0.284, 0.465] | 0.796 |
| str_mean_dd3 ~ 1 | 0.375 | 0.046 | 8.106 | < .001 | *** | [0.284, 0.465] | 0.796 |
| STR2 ~ 1 | 0.000 | 0.000 |  |  |  | [0.000, 0.000] | 0.000 |
| dSTR ~ 1 | -0.015 | 0.048 | -0.315 | .753 |  | [-0.109, 0.079] | -0.033 |
| age_w2_c ~ 1 | -0.000 | 0.331 | -0.000 | 1.000 |  | [-0.648, 0.648] | -0.000 |
| white ~ 1 | 0.868 | 0.011 | 80.331 | < .001 | *** | [0.847, 0.889] | 2.562 |
| edu_college ~ 1 | 0.442 | 0.016 | 27.958 | < .001 | *** | [0.411, 0.473] | 0.890 |
| sex_clean ~ 1 | 0.572 | 0.016 | 36.327 | < .001 | *** | [0.541, 0.603] | 1.156 |
| chronic_c ~ 1 | -0.003 | 0.073 | -0.035 | .972 |  | [-0.145, 0.140] | -0.001 |
| STR3 ~ 1 | 0.000 | 0.000 |  |  |  | [0.000, 0.000] | 0.000 |

## 5.7. R-squared for endogenous variables

| Variable | R2 |
| --- | --- |
| EF3 | 1.000 |
| EF2 | 0.476 |
| dEF | 0.078 |
| POS3 | 1.000 |
| POS2 | 0.077 |
| dPOS | 0.013 |
| STR3 | 1.000 |
| STR2 | 0.104 |
| dSTR | 0.004 |
| pos_mean_dd2 | 1.000 |
| pos_mean_dd3 | 1.000 |
| str_mean_dd2 | 1.000 |
| str_mean_dd3 | 1.000 |
| M2 DIG BACKW | 0.179 |
| M2 CAT FLUEN | 0.253 |
| M2 STOP & GO | 0.309 |
| B3TNSTOT | 0.467 |
| M2 30S AND COUNT | 0.544 |
| M3 DIG BACKW | 0.195 |
| M3 CAT FLUEN | 0.296 |
| M3 STOP & GO | 0.144 |
| C3TNSTOT | 0.504 |
| M3 30S AND COUNT | 0.564 |

# 6. Full Model: Episodic Memory without covariates

2025-10-07 13:37

## 6.1. Model specifications

| Specification | Value |
| --- | --- |
| Estimator |  |
| Missing handling | ml |
| # Groups | 1 |
| Group labels | (none) |
| N (total) | 988 |

## 6.2. Global fit indices

| Measure | Value |
| --- | --- |
| Chi-square | 8.806 |
| df | 10 |
| p-value | .551 |
| CFI | 1.000 |
| TLI | 1.001 |
| RMSEA | 0.000 |
| RMSEA 90% CI lower | 0.000 |
| RMSEA 90% CI upper | 0.031 |
| RMSEA p-close |  |
| SRMR | 0.011 |
| AIC | 21356.713 |
| BIC | 21523.167 |

## 6.3. Factor loadings (std.all)

| Term | Est | SE | z-value | p | sig | CI | Std (all) |
| --- | --- | --- | --- | --- | --- | --- | --- |
| EM2 =~ M2 IMM RECALL | 1.000 | 0.000 |  |  |  | [1.000, 1.000] | 0.947 |
| EM2 =~ M2 DEL RECALL | 1.000 | 0.000 |  |  |  | [1.000, 1.000] | 0.823 |
| EM3 =~ M3 IMM RECALL | 1.000 | 0.000 |  |  |  | [1.000, 1.000] | 0.959 |
| EM3 =~ M3 DEL RECALL | 1.000 | 0.000 |  |  |  | [1.000, 1.000] | 0.840 |
| dEM =~ EM3 | 1.000 | 0.000 |  |  |  | [1.000, 1.000] | 0.876 |
| POS2 =~ pos_mean_dd2 | 1.000 | 0.000 |  |  |  | [1.000, 1.000] | 1.000 |
| POS3 =~ pos_mean_dd3 | 1.000 | 0.000 |  |  |  | [1.000, 1.000] | 1.000 |
| dPOS =~ POS3 | 1.000 | 0.000 |  |  |  | [1.000, 1.000] | 0.854 |
| STR2 =~ str_mean_dd2 | 1.000 | 0.000 |  |  |  | [1.000, 1.000] | 1.000 |
| STR3 =~ str_mean_dd3 | 1.000 | 0.000 |  |  |  | [1.000, 1.000] | 1.000 |
| dSTR =~ STR3 | 1.000 | 0.000 |  |  |  | [1.000, 1.000] | 0.962 |

## 6.4. Regressions (std.all)

| Term | Est | SE | z-value | p | sig | CI | Std (all) |
| --- | --- | --- | --- | --- | --- | --- | --- |
| EM3 ~ EM2 | 1.000 | 0.000 |  |  |  | [1.000, 1.000] | 0.927 |
| POS3 ~ POS2 | 1.000 | 0.000 |  |  |  | [1.000, 1.000] | 0.915 |
| STR3 ~ STR2 | 1.000 | 0.000 |  |  |  | [1.000, 1.000] | 1.001 |

## 6.5. Variances & covariances (std.all)

| Term | Est | SE | z-value | p | sig | CI | Std (all) |
| --- | --- | --- | --- | --- | --- | --- | --- |
| M2 IMM RECALL ~~ M3 IMM RECALL | -0.260 | 0.088 | -2.943 | .003 | ** | [-0.434, -0.087] | -0.570 |
| M2 DEL RECALL ~~ M3 DEL RECALL | 0.799 | 0.130 | 6.142 | < .001 | *** | [0.544, 1.054] | 0.395 |
| EM3 ~~ dEM | 0.000 | 0.000 |  |  |  | [0.000, 0.000] |  |
| Var(EM3) | 0.000 | 0.000 |  |  |  | [0.000, 0.000] | 0.000 |
| Var(EM2) | 4.202 | 0.242 | 17.371 | < .001 | *** | [3.728, 4.677] | 1.000 |
| Var(dEM) | 3.758 | 0.268 | 14.000 | < .001 | *** | [3.232, 4.284] | 1.000 |
| EM2 ~~ dEM | -1.534 | 0.186 | -8.244 | < .001 | *** | [-1.899, -1.169] | -0.386 |
| POS3 ~~ dPOS | 0.000 | 0.000 |  |  |  | [0.000, 0.000] |  |
| Var(POS3) | 0.000 | 0.000 |  |  |  | [0.000, 0.000] | 0.000 |
| Var(POS2) | 0.470 | 0.024 | 19.410 | < .001 | *** | [0.422, 0.517] | 1.000 |
| Var(dPOS) | 0.410 | 0.023 | 18.085 | < .001 | *** | [0.365, 0.454] | 1.000 |
| POS2 ~~ dPOS | -0.159 | 0.017 | -9.220 | < .001 | *** | [-0.193, -0.125] | -0.363 |
| STR3 ~~ dSTR | 0.000 | 0.000 |  |  |  | [0.000, 0.000] |  |
| Var(STR3) | 0.000 | 0.000 |  |  |  | [0.000, 0.000] | 0.000 |
| Var(STR2) | 0.222 | 0.018 | 12.183 | < .001 | *** | [0.186, 0.258] | 1.000 |
| Var(dSTR) | 0.205 | 0.015 | 13.472 | < .001 | *** | [0.175, 0.235] | 1.000 |
| STR2 ~~ dSTR | -0.103 | 0.013 | -7.776 | < .001 | *** | [-0.129, -0.077] | -0.482 |
| EM2 ~~ POS2 | 0.175 | 0.047 | 3.761 | < .001 | *** | [0.084, 0.266] | 0.125 |
| EM2 ~~ STR2 | 0.084 | 0.032 | 2.656 | .008 | ** | [0.022, 0.147] | 0.087 |
| POS2 ~~ STR2 | 0.143 | 0.014 | 10.030 | < .001 | *** | [0.115, 0.172] | 0.444 |
| dEM ~~ dPOS | 0.037 | 0.046 | 0.808 | .419 |  | [-0.053, 0.127] | 0.030 |
| dEM ~~ dSTR | 0.007 | 0.033 | 0.205 | .838 |  | [-0.058, 0.072] | 0.008 |
| dPOS ~~ dSTR | 0.039 | 0.012 | 3.178 | .001 | ** | [0.015, 0.064] | 0.136 |
| EM2 ~~ dPOS | 0.025 | 0.044 | 0.569 | .569 |  | [-0.061, 0.111] | 0.019 |
| EM2 ~~ dSTR | -0.047 | 0.031 | -1.484 | .138 |  | [-0.108, 0.015] | -0.050 |
| dEM ~~ POS2 | 0.007 | 0.049 | 0.144 | .886 |  | [-0.088, 0.102] | 0.005 |
| POS2 ~~ dSTR | -0.051 | 0.012 | -4.298 | < .001 | *** | [-0.074, -0.028] | -0.164 |
| dEM ~~ STR2 | 0.066 | 0.034 | 1.953 | .051 |  | [-0.000, 0.132] | 0.072 |
| dPOS ~~ STR2 | -0.036 | 0.013 | -2.810 | .005 | ** | [-0.060, -0.011] | -0.118 |
| Var(pos_mean_dd2) | 0.000 | 0.000 |  |  |  | [0.000, 0.000] | 0.000 |
| Var(pos_mean_dd3) | 0.000 | 0.000 |  |  |  | [0.000, 0.000] | 0.000 |
| Var(str_mean_dd2) | 0.000 | 0.000 |  |  |  | [0.000, 0.000] | 0.000 |
| Var(str_mean_dd3) | 0.000 | 0.000 |  |  |  | [0.000, 0.000] | 0.000 |
| Var(M2 IMM RECALL) | 0.486 | 0.108 | 4.486 | < .001 | *** | [0.274, 0.698] | 0.104 |
| Var(M2 DEL RECALL) | 2.008 | 0.159 | 12.615 | < .001 | *** | [1.696, 2.320] | 0.323 |
| Var(M3 IMM RECALL) | 0.429 | 0.115 | 3.725 | < .001 | *** | [0.203, 0.654] | 0.081 |
| Var(M3 DEL RECALL) | 2.035 | 0.155 | 13.156 | < .001 | *** | [1.731, 2.338] | 0.294 |

## 6.6. Intercepts / means

| Term | Est | SE | z-value | p | sig | CI | Std (all) |
| --- | --- | --- | --- | --- | --- | --- | --- |
| M2 IMM RECALL ~ 1 | 7.050 | 0.068 | 104.448 | < .001 | *** | [6.918, 7.183] | 3.256 |
| M3 IMM RECALL ~ 1 | 7.050 | 0.068 | 104.448 | < .001 | *** | [6.918, 7.183] | 3.056 |
| M2 DEL RECALL ~ 1 | 4.731 | 0.076 | 62.003 | < .001 | *** | [4.581, 4.880] | 1.898 |
| M3 DEL RECALL ~ 1 | 4.731 | 0.076 | 62.003 | < .001 | *** | [4.581, 4.880] | 1.797 |
| EM2 ~ 1 | 0.000 | 0.000 |  |  |  | [0.000, 0.000] | 0.000 |
| dEM ~ 1 | -0.116 | 0.068 | -1.700 | .089 |  | [-0.251, 0.018] | -0.060 |
| EM3 ~ 1 | 0.000 | 0.000 |  |  |  | [0.000, 0.000] | 0.000 |
| pos_mean_dd2 ~ 1 | 1.173 | 0.022 | 53.784 | < .001 | *** | [1.130, 1.215] | 1.711 |
| pos_mean_dd3 ~ 1 | 1.173 | 0.022 | 53.784 | < .001 | *** | [1.130, 1.215] | 1.565 |
| POS2 ~ 1 | 0.000 | 0.000 |  |  |  | [0.000, 0.000] | 0.000 |
| dPOS ~ 1 | 0.317 | 0.021 | 15.337 | < .001 | *** | [0.277, 0.358] | 0.496 |
| POS3 ~ 1 | 0.000 | 0.000 |  |  |  | [0.000, 0.000] | 0.000 |
| str_mean_dd2 ~ 1 | 0.579 | 0.015 | 38.605 | < .001 | *** | [0.549, 0.608] | 1.228 |
| str_mean_dd3 ~ 1 | 0.579 | 0.015 | 38.605 | < .001 | *** | [0.549, 0.608] | 1.229 |
| STR2 ~ 1 | 0.000 | 0.000 |  |  |  | [0.000, 0.000] | 0.000 |
| dSTR ~ 1 | -0.068 | 0.015 | -4.663 | < .001 | *** | [-0.096, -0.039] | -0.149 |
| STR3 ~ 1 | 0.000 | 0.000 |  |  |  | [0.000, 0.000] | 0.000 |

## 6.7. R-squared for endogenous variables

| Variable | R2 |
| --- | --- |
| EM3 | 1.000 |
| POS3 | 1.000 |
| STR3 | 1.000 |
| pos_mean_dd2 | 1.000 |
| pos_mean_dd3 | 1.000 |
| str_mean_dd2 | 1.000 |
| str_mean_dd3 | 1.000 |
| M2 IMM RECALL | 0.896 |
| M2 DEL RECALL | 0.677 |
| M3 IMM RECALL | 0.919 |
| M3 DEL RECALL | 0.706 |

# 7. Full Model: Episodic Memory with covariates

## 7.1. Model specifications

| Specification | Value |
| --- | --- |
| Estimator |  |
| Missing handling | ml |
| # Groups | 1 |
| Group labels | (none) |
| N (total) | 988 |

## 7.2. Global fit indices

| Measure | Value |
| --- | --- |
| Chi-square | 44.346 |
| df | 20 |
| p-value | .001 |
| CFI | 0.994 |
| TLI | 0.975 |
| RMSEA | 0.035 |
| RMSEA 90% CI lower | 0.021 |
| RMSEA 90% CI upper | 0.049 |
| RMSEA p-close |  |
| SRMR | 0.013 |
| AIC | 36119.719 |
| BIC | 36530.956 |

## 7.3. Factor loadings (std.all)

| Term | Est | SE | z-value | p | sig | CI | Std (all) |
| --- | --- | --- | --- | --- | --- | --- | --- |
| EM2 =~ M2 IMM RECALL | 1.000 | 0.000 |  |  |  | [1.000, 1.000] | 0.935 |
| EM2 =~ M2 DEL RECALL | 1.000 | 0.000 |  |  |  | [1.000, 1.000] | 0.827 |
| EM3 =~ M3 IMM RECALL | 1.000 | 0.000 |  |  |  | [1.000, 1.000] | 0.944 |
| EM3 =~ M3 DEL RECALL | 1.000 | 0.000 |  |  |  | [1.000, 1.000] | 0.847 |
| dEM =~ EM3 | 1.000 | 0.000 |  |  |  | [1.000, 1.000] | 0.886 |
| POS2 =~ pos_mean_dd2 | 1.000 | 0.000 |  |  |  | [1.000, 1.000] | 1.000 |
| POS3 =~ pos_mean_dd3 | 1.000 | 0.000 |  |  |  | [1.000, 1.000] | 1.000 |
| dPOS =~ POS3 | 1.000 | 0.000 |  |  |  | [1.000, 1.000] | 0.857 |
| STR2 =~ str_mean_dd2 | 1.000 | 0.000 |  |  |  | [1.000, 1.000] | 1.000 |
| STR3 =~ str_mean_dd3 | 1.000 | 0.000 |  |  |  | [1.000, 1.000] | 1.000 |
| dSTR =~ STR3 | 1.000 | 0.000 |  |  |  | [1.000, 1.000] | 0.962 |

## 7.4. Regressions (std.all)

| Term | Est | SE | z-value | p | sig | CI | Std (all) |
| --- | --- | --- | --- | --- | --- | --- | --- |
| EM3 ~ EM2 | 1.000 | 0.000 |  |  |  | [1.000, 1.000] | 0.928 |
| POS3 ~ POS2 | 1.000 | 0.000 |  |  |  | [1.000, 1.000] | 0.916 |
| STR3 ~ STR2 | 1.000 | 0.000 |  |  |  | [1.000, 1.000] | 1.000 |
| EM2 ~ age_w2_c | -0.036 | 0.006 | -5.749 | < .001 | *** | [-0.049, -0.024] | -0.186 |
| EM2 ~ white | 0.715 | 0.199 | 3.594 | < .001 | *** | [0.325, 1.104] | 0.119 |
| EM2 ~ edu_college | 0.725 | 0.128 | 5.648 | < .001 | *** | [0.473, 0.976] | 0.177 |
| EM2 ~ sex_clean | 1.176 | 0.128 | 9.195 | < .001 | *** | [0.925, 1.427] | 0.287 |
| EM2 ~ chronic_c | -0.045 | 0.027 | -1.657 | .098 |  | [-0.098, 0.008] | -0.050 |
| dEM ~ age_w2_c | -0.022 | 0.007 | -3.231 | .001 | ** | [-0.035, -0.009] | -0.116 |
| dEM ~ white | -0.181 | 0.192 | -0.944 | .345 |  | [-0.556, 0.195] | -0.032 |
| dEM ~ edu_college | -0.017 | 0.145 | -0.114 | .909 |  | [-0.300, 0.267] | -0.004 |
| dEM ~ sex_clean | 0.246 | 0.138 | 1.780 | .075 |  | [-0.025, 0.518] | 0.063 |
| dEM ~ chronic_c | -0.062 | 0.030 | -2.068 | .039 | * | [-0.121, -0.003] | -0.073 |
| POS2 ~ age_w2_c | 0.005 | 0.002 | 2.249 | .025 | * | [0.001, 0.009] | 0.072 |
| POS2 ~ white | 0.118 | 0.065 | 1.810 | .070 |  | [-0.010, 0.245] | 0.058 |
| POS2 ~ edu_college | 0.331 | 0.044 | 7.587 | < .001 | *** | [0.245, 0.416] | 0.240 |
| POS2 ~ sex_clean | 0.181 | 0.043 | 4.177 | < .001 | *** | [0.096, 0.266] | 0.131 |
| POS2 ~ chronic_c | -0.000 | 0.010 | -0.008 | .993 |  | [-0.019, 0.019] | -0.000 |
| dPOS ~ age_w2_c | 0.005 | 0.002 | 2.419 | .016 | * | [0.001, 0.009] | 0.083 |
| dPOS ~ white | 0.132 | 0.060 | 2.200 | .028 | * | [0.014, 0.249] | 0.070 |
| dPOS ~ edu_college | 0.018 | 0.045 | 0.392 | .695 |  | [-0.070, 0.106] | 0.014 |
| dPOS ~ sex_clean | 0.014 | 0.043 | 0.323 | .746 |  | [-0.070, 0.098] | 0.011 |
| dPOS ~ chronic_c | -0.008 | 0.010 | -0.757 | .449 |  | [-0.028, 0.012] | -0.028 |
| STR2 ~ age_w2_c | -0.008 | 0.001 | -6.170 | < .001 | *** | [-0.011, -0.006] | -0.185 |
| STR2 ~ white | 0.086 | 0.044 | 1.952 | .051 |  | [-0.000, 0.173] | 0.062 |
| STR2 ~ edu_college | 0.188 | 0.032 | 5.869 | < .001 | *** | [0.125, 0.250] | 0.198 |
| STR2 ~ sex_clean | 0.080 | 0.029 | 2.736 | .006 | ** | [0.023, 0.138] | 0.084 |
| STR2 ~ chronic_c | 0.040 | 0.010 | 3.962 | < .001 | *** | [0.020, 0.059] | 0.192 |
| dSTR ~ age_w2_c | -0.000 | 0.002 | -0.252 | .801 |  | [-0.003, 0.003] | -0.009 |
| dSTR ~ white | -0.013 | 0.043 | -0.309 | .757 |  | [-0.098, 0.071] | -0.010 |
| dSTR ~ edu_college | -0.018 | 0.031 | -0.598 | .550 |  | [-0.079, 0.042] | -0.020 |
| dSTR ~ sex_clean | -0.058 | 0.030 | -1.923 | .055 |  | [-0.118, 0.001] | -0.064 |
| dSTR ~ chronic_c | 0.004 | 0.007 | 0.523 | .601 |  | [-0.011, 0.019] | 0.020 |

## 7.5. Variances & covariances (std.all)

| Term | Est | SE | z-value | p | sig | CI | Std (all) |
| --- | --- | --- | --- | --- | --- | --- | --- |
| M2 IMM RECALL ~~ M3 IMM RECALL | -0.136 | 0.083 | -1.646 | .100 |  | [-0.298, 0.026] | -0.232 |
| M2 DEL RECALL ~~ M3 DEL RECALL | 0.675 | 0.124 | 5.436 | < .001 | *** | [0.432, 0.918] | 0.357 |
| EM3 ~~ dEM | 0.000 | 0.000 |  |  |  | [0.000, 0.000] |  |
| Var(EM3) | 0.000 | 0.000 |  |  |  | [0.000, 0.000] | 0.000 |
| Var(EM2) | 3.472 | 0.217 | 15.988 | < .001 | *** | [3.046, 3.897] | 0.843 |
| Var(dEM) | 3.662 | 0.263 | 13.922 | < .001 | *** | [3.146, 4.177] | 0.975 |
| EM2 ~~ dEM | -1.698 | 0.183 | -9.266 | < .001 | *** | [-2.057, -1.339] | -0.476 |
| POS3 ~~ dPOS | 0.000 | 0.000 |  |  |  | [0.000, 0.000] |  |
| Var(POS3) | 0.000 | 0.000 |  |  |  | [0.000, 0.000] | 0.000 |
| Var(POS2) | 0.434 | 0.023 | 18.460 | < .001 | *** | [0.388, 0.480] | 0.923 |
| Var(dPOS) | 0.405 | 0.023 | 17.902 | < .001 | *** | [0.361, 0.450] | 0.987 |
| POS2 ~~ dPOS | -0.167 | 0.017 | -10.069 | < .001 | *** | [-0.200, -0.135] | -0.399 |
| STR3 ~~ dSTR | 0.000 | 0.000 |  |  |  | [0.000, 0.000] |  |
| Var(STR3) | 0.000 | 0.000 |  |  |  | [0.000, 0.000] | 0.000 |
| Var(STR2) | 0.199 | 0.016 | 12.379 | < .001 | *** | [0.167, 0.230] | 0.896 |
| Var(dSTR) | 0.204 | 0.015 | 13.604 | < .001 | *** | [0.175, 0.234] | 0.996 |
| STR2 ~~ dSTR | -0.102 | 0.012 | -8.125 | < .001 | *** | [-0.126, -0.077] | -0.504 |
| EM2 ~~ POS2 | 0.078 | 0.041 | 1.936 | .053 |  | [-0.001, 0.158] | 0.064 |
| EM2 ~~ STR2 | -0.000 | 0.028 | -0.014 | .989 |  | [-0.055, 0.055] | -0.000 |
| POS2 ~~ STR2 | 0.129 | 0.013 | 10.146 | < .001 | *** | [0.104, 0.153] | 0.438 |
| dEM ~~ dPOS | 0.048 | 0.046 | 1.055 | .292 |  | [-0.042, 0.138] | 0.040 |
| dEM ~~ dSTR | 0.011 | 0.033 | 0.351 | .726 |  | [-0.053, 0.076] | 0.013 |
| dPOS ~~ dSTR | 0.040 | 0.012 | 3.244 | .001 | ** | [0.016, 0.064] | 0.139 |
| EM2 ~~ dPOS | 0.025 | 0.039 | 0.635 | .526 |  | [-0.051, 0.100] | 0.021 |
| EM2 ~~ dSTR | -0.028 | 0.029 | -0.965 | .334 |  | [-0.084, 0.029] | -0.033 |
| dEM ~~ POS2 | 0.012 | 0.045 | 0.273 | .785 |  | [-0.076, 0.101] | 0.010 |
| POS2 ~~ dSTR | -0.047 | 0.011 | -4.268 | < .001 | *** | [-0.068, -0.025] | -0.157 |
| dEM ~~ STR2 | 0.056 | 0.032 | 1.743 | .081 |  | [-0.007, 0.118] | 0.065 |
| dPOS ~~ STR2 | -0.033 | 0.012 | -2.689 | .007 | ** | [-0.057, -0.009] | -0.116 |
| Var(pos_mean_dd2) | 0.000 | 0.000 |  |  |  | [0.000, 0.000] | 0.000 |
| Var(pos_mean_dd3) | 0.000 | 0.000 |  |  |  | [0.000, 0.000] | 0.000 |
| Var(str_mean_dd2) | 0.000 | 0.000 |  |  |  | [0.000, 0.000] | 0.000 |
| Var(str_mean_dd3) | 0.000 | 0.000 |  |  |  | [0.000, 0.000] | 0.000 |
| age_w2_c ~~ white | 0.158 | 0.115 | 1.370 | .171 |  | [-0.068, 0.383] | 0.045 |
| age_w2_c ~~ edu_college | -0.217 | 0.163 | -1.327 | .185 |  | [-0.537, 0.104] | -0.042 |
| age_w2_c ~~ sex_clean | -0.017 | 0.162 | -0.102 | .919 |  | [-0.334, 0.301] | -0.003 |
| age_w2_c ~~ chronic_c | 3.796 | 0.738 | 5.142 | < .001 | *** | [2.349, 5.243] | 0.161 |
| white ~~ edu_college | 0.023 | 0.005 | 4.467 | < .001 | *** | [0.013, 0.033] | 0.136 |
| white ~~ sex_clean | -0.006 | 0.005 | -1.083 | .279 |  | [-0.016, 0.005] | -0.034 |
| white ~~ chronic_c | -0.046 | 0.028 | -1.658 | .097 |  | [-0.100, 0.008] | -0.060 |
| edu_college ~~ sex_clean | -0.034 | 0.008 | -4.304 | < .001 | *** | [-0.049, -0.018] | -0.136 |
| edu_college ~~ chronic_c | -0.145 | 0.035 | -4.133 | < .001 | *** | [-0.214, -0.076] | -0.129 |
| sex_clean ~~ chronic_c | 0.181 | 0.035 | 5.239 | < .001 | *** | [0.114, 0.249] | 0.161 |
| Var(M2 IMM RECALL) | 0.592 | 0.106 | 5.571 | < .001 | *** | [0.384, 0.800] | 0.126 |
| Var(M2 DEL RECALL) | 1.904 | 0.155 | 12.261 | < .001 | *** | [1.600, 2.208] | 0.316 |
| Var(M3 IMM RECALL) | 0.584 | 0.111 | 5.253 | < .001 | *** | [0.366, 0.802] | 0.109 |
| Var(M3 DEL RECALL) | 1.877 | 0.145 | 12.955 | < .001 | *** | [1.593, 2.161] | 0.282 |
| Var(age_w2_c) | 108.112 | 3.986 | 27.122 | < .001 | *** | [100.299, 115.925] | 1.000 |
| Var(white) | 0.115 | 0.008 | 14.432 | < .001 | *** | [0.099, 0.130] | 1.000 |
| Var(edu_college) | 0.247 | 0.002 | 134.731 | < .001 | *** | [0.243, 0.250] | 1.000 |
| Var(sex_clean) | 0.245 | 0.002 | 108.214 | < .001 | *** | [0.240, 0.249] | 1.000 |
| Var(chronic_c) | 5.158 | 0.448 | 11.514 | < .001 | *** | [4.280, 6.036] | 1.000 |

## 7.6. Intercepts / means

| Term | Est | SE | z-value | p | sig | CI | Std (all) |
| --- | --- | --- | --- | --- | --- | --- | --- |
| M2 IMM RECALL ~ 1 | 5.437 | 0.207 | 26.289 | < .001 | *** | [5.031, 5.842] | 2.505 |
| M3 IMM RECALL ~ 1 | 5.437 | 0.207 | 26.289 | < .001 | *** | [5.031, 5.842] | 2.347 |
| M2 DEL RECALL ~ 1 | 3.118 | 0.209 | 14.946 | < .001 | *** | [2.709, 3.526] | 1.270 |
| M3 DEL RECALL ~ 1 | 3.118 | 0.209 | 14.946 | < .001 | *** | [2.709, 3.526] | 1.208 |
| EM2 ~ 1 | 0.000 | 0.000 |  |  |  | [0.000, 0.000] | 0.000 |
| dEM ~ 1 | -0.094 | 0.194 | -0.484 | .628 |  | [-0.475, 0.287] | -0.049 |
| EM3 ~ 1 | 0.000 | 0.000 |  |  |  | [0.000, 0.000] | 0.000 |
| pos_mean_dd2 ~ 1 | 0.821 | 0.067 | 12.260 | < .001 | *** | [0.689, 0.952] | 1.198 |
| pos_mean_dd3 ~ 1 | 0.821 | 0.067 | 12.260 | < .001 | *** | [0.689, 0.952] | 1.097 |
| POS2 ~ 1 | 0.000 | 0.000 |  |  |  | [0.000, 0.000] | 0.000 |
| dPOS ~ 1 | 0.188 | 0.062 | 3.019 | .003 | ** | [0.066, 0.309] | 0.293 |
| POS3 ~ 1 | 0.000 | 0.000 |  |  |  | [0.000, 0.000] | 0.000 |
| str_mean_dd2 ~ 1 | 0.375 | 0.046 | 8.104 | < .001 | *** | [0.284, 0.466] | 0.796 |
| str_mean_dd3 ~ 1 | 0.375 | 0.046 | 8.104 | < .001 | *** | [0.284, 0.466] | 0.796 |
| STR2 ~ 1 | 0.000 | 0.000 |  |  |  | [0.000, 0.000] | 0.000 |
| dSTR ~ 1 | -0.015 | 0.048 | -0.312 | .755 |  | [-0.109, 0.079] | -0.033 |
| age_w2_c ~ 1 | 0.000 | 0.331 | 0.000 | 1.000 |  | [-0.648, 0.648] | 0.000 |
| white ~ 1 | 0.868 | 0.011 | 80.483 | < .001 | *** | [0.847, 0.889] | 2.564 |
| edu_college ~ 1 | 0.442 | 0.016 | 27.946 | < .001 | *** | [0.411, 0.473] | 0.890 |
| sex_clean ~ 1 | 0.572 | 0.016 | 36.327 | < .001 | *** | [0.541, 0.603] | 1.156 |
| chronic_c ~ 1 | -0.001 | 0.073 | -0.015 | .988 |  | [-0.144, 0.142] | -0.000 |
| STR3 ~ 1 | 0.000 | 0.000 |  |  |  | [0.000, 0.000] | 0.000 |

## 7.7. R-squared for endogenous variables

| Variable | R2 |
| --- | --- |
| EM3 | 1.000 |
| EM2 | 0.157 |
| dEM | 0.025 |
| POS3 | 1.000 |
| POS2 | 0.077 |
| dPOS | 0.013 |
| STR3 | 1.000 |
| STR2 | 0.104 |
| dSTR | 0.004 |
| pos_mean_dd2 | 1.000 |
| pos_mean_dd3 | 1.000 |
| str_mean_dd2 | 1.000 |
| str_mean_dd3 | 1.000 |
| M2 IMM RECALL | 0.874 |
| M2 DEL RECALL | 0.684 |
| M3 IMM RECALL | 0.891 |
| M3 DEL RECALL | 0.718 |

# 8. Discussion of covariate effects

## 8.1. Covariate Effects on Executive Functioning and Daily Events

Several demographic and health-related covariates were associated with baseline levels and change in executive functioning, positive events, and stressors. At baseline, older age, more chronic conditions, and being a woman (vs. man) were each associated with lower executive functioning, whereas identifying as white and having a college degree were associated with higher executive functioning. For change in executive functioning, only older age predicted greater decline over time; the remaining covariates did not significantly predict change. Turning to daily events, older age, having a college degree, and being a woman were associated with reporting more positive events at baseline, and older age and white race were associated with greater increases in positive events over time. Higher stressor levels at baseline were associated with younger age, college education, being a woman, and greater number of chronic conditions; no covariate significantly predicted change in stressors.

## 8.2. Covariate Effects on Episodic Memory and Daily Events

The pattern of covariate effects on episodic memory largely mirrored that observed for executive functioning. At baseline, older age was associated with lower episodic memory, while identifying as white, having a college degree, and being a woman were each associated with higher episodic memory; chronic conditions showed only a marginal negative association. For change in episodic memory, older age and greater number of chronic conditions were each associated with greater decline over time, whereas race, education, and gender did not significantly predict change. The covariate effects on positive events and stressors were consistent with those reported for the executive functioning model.
